# Characteristics of the Rest and Activity Patterns in Patients With Mild to Moderate Parkinson's Syndrome

**DOI:** 10.1111/psyg.70047

**Published:** 2025-05-08

**Authors:** Kaori Ono, Yu Kume

**Affiliations:** ^1^ Graduate School of Medicine, Doctoral Course in Health Sciences Akita University Akita Japan; ^2^ Akita Prefectural Center for Rehabilitation and Psychiatric Medicine Daisen Japan; ^3^ Graduate School of Medicine, Department of Occupational Therapy Akita University Akita Japan

**Keywords:** actigraph, Parkinson's syndrome, rest‐activity rhythm

## Abstract

**Background:**

Few studies have examined the characteristics of the decline in physical activity levels in Parkinson's disease (PD) patients from the perspective of a 24‐h behavioural profile, including sleep.

**Objective:**

To identify factors related to the rest and activity patterns in PD patients by assessing the Rest‐Activity Rhythm.

**Methods:**

The participants were 20 patients with Parkinson's syndrome (PS group) and 20 healthy elderly community residents (control group). The nonparametric rest‐activity rhythm parameters were measured by a wristwatch‐type activity monitor worn by the study subjects on the non‐dominant hand continuously for at least 5 days. To assess the specific symptoms of PS in the patients, the scores on the Movement Disorder Society‐Sponsored Revision of the Unified Parkinson's Disease Rating Scale (MDS‐UPDRS) were collected from the medical records.

**Results:**

In the PS group, a negative correlation was found between the relative amplitude and score for part I of the MDS‐UPDRS, that is, non‐motor symptoms (*r* = −0.51, *p* < 0.05). Additionally, binomial logistic regression analysis revealed two regression models, with Model I showing a significant association with the highest physical activity level over the 24‐h profile (odds ratio, 0.96; 95% confidence interval [95% CI], 0.94–0.99; *p* = 0.002) and Model II showing a significant association with a fragmented rhythm (odds ratio, 1.04; 95% CI, 1.01–1.07; *p* = 0.004).

**Conclusion:**

These results suggest that rehabilitation of PD patients should include increasing their physical activity levels while minimising intermittency of the rest and activity patterns, and that attention should be paid to non‐motor symptoms in addition to motor symptoms.

## 1 Introduction

Parkinson's disease (PD) is a chronic progressive disease that is characterised by motor symptoms such as tremor, muscle rigidity, bradykinesia and postural dysreflexia, and non‐motor symptoms such as tremor, sleep disturbances and psychiatric and cognitive symptoms. In addition, aging and environmental changes also lead to a gradual decline in the physical activity levels in these patients, resulting in a decrease in the activities of daily living (ADL) and quality of life (QoL). It is generally reported that the physical activity levels in PD patients decrease with increasing disease severity [1]. Cavanaugh et al. reported that PD patients with Hoehn and Yahr (H&Y) severity Class I–III, who initially have a relatively high level of independence, showed a significant decline in physical activity levels at 1 year [2]. In addition, Speelman et al. pointed out that factors such as sedentary lifestyle, falls, depression and apathy also contribute to the decline in physical activity levels in PD patients [3]. Furthermore, Van Someren et al. evaluated the motor symptoms, circadian rhythms and nocturnal psychiatric symptoms in patients with neurodegenerative diseases such as PD and Alzheimer's disease using the actigraph and demonstrated the validity of monitoring in these patients [4]. The goal of rehabilitative care for PD patients with these characteristics is to delay the worsening of functional disability as much as possible and to maintain QoL [5]. Rehabilitative care should focus not only on the physical and mental functions, but also on the social activity and participation of these patients.

The physical activity levels in PD patients have been reported to show chronological changes during the day, varying with the time of day [6]. However, few studies have examined the characteristics of the reduced physical activity levels in PD patients from the perspective of the 24‐h behavioural profile of these patients, including physical activities in the daytime and sleep at night. For appropriate treatment of PD patients, it is necessary to accurately capture the fluctuation pattern of the symptoms, although at present, we can only rely on subjective reports from the patients, such as symptom diaries.

The purpose of this study was to clarify the physical activity levels of patients with Parkinson's syndrome (PS) in terms of the rest and activity patterns using the Rest‐Activity Rhythm (RAR) parameters, which has been widely used to objectively determine the daily activity levels and the length of sleep and wake times, and to identify factors of PS related to the RAR. Physical activity is defined here as ‘any bodily movement produced by contractile activity of the skeletal muscles’ [7] and is considered a broad concept that includes daily activities such as housework, mobility and work in daily life. Based on the above, we expect that visualisation of RAR patterns of patients with PS would enable us to provide specific lifestyle guidance, prevent inactive lifestyles and promote active lifestyles.

## 2 Methods

### 2.1 Participants

A total of 20 patients with PS who were admitted to the Akita Rehabilitation and Psychiatric Center between February 2023 and July 2024 were receiving medication and rehabilitative care. In this study, in addition to PD, the following diseases characterised by parkinsonian symptoms were included: multiple system atrophy (MSA), progressive supranuclear palsy (PSP) and corticobasal degeneration (CBD) (those classified as V according to the H&Y severity classification [8]). Patients with severe psychiatric symptoms or significant cognitive impairment were excluded from the study. The control group consisted of 20 healthy community‐dwelling elderly persons aged 65 years or more who participated in a community dementia prevention program between July 2022 and January 2024; those who had a central nervous system disease or required assistance with daily living were excluded. This study was approved by the ethics committee of the Faculty of Medicine, Akita University (approval No. 2924).

### 2.2 Demographic Information

The following data of the patients of the PS group were recorded for this study: age, sex, disease name, time elapsed since diagnosis, H&Y severity classification as a disease‐specific assessment, score on the Movement Disorder Society‐Sponsored Revision of the Unified Parkinson's Disease Rating Scale (MDS‐UPDRS) [9, 10, 11], score on the Clinical Dementia Rating scale (CDR) [12] to assess cognitive function, and score on the Japanese version of the Functional Independence Measure (FIM) to assess the ADLs [13, 14, 15]. The Movement Disorder Society‐Sponsored Revision of the Unified Parkinson's Disease Rating Scale (MDS‐UPDRS) is a scale used to assess the severity of PS and comprises four parts: Part I: Non‐Motor Experiences of Daily Living (13 items, score range 0–52); Part II: Motor Experiences of Daily Living; Part III: Motor Examination (18 items, score range 0–132); Part IV: Motor Complications (6 items, score range 0–24). The total score therefore ranges from 0 to 260, with higher scores indicating more severe disease conditions. On the other hand, older‐community‐dwelling adults were assessed using the CDR and FIM to assess the ADLs.

### 2.3 Non‐Parametric Rest‐Activity Rhythm (RAR) Parameters

Rest‐activity patterns were measured using a wrist‐worn activity monitor, the Actiwatch Spectrum plus, which the subjects wore continuously on the non‐dominant hand for at least 5 days. A non‐parametric circadian resting‐activity rhythm analysis [16] was performed using the collected actigraph data, and the inter‐day stability (IS), intraday variability (IV), relative amplitude (RA), average activity of the least active 5‐h period over a 24 h period (L5) and the average activity of the most active 10‐h period over a 24 h period (M10) were calculated. IS indicates the regularity of the rest‐activity patterns, IV reflects continuity of physical activity and RA means the degree of balance between rest and activity [17].

### 2.4 Data Analysis

An unpaired *t*‐test, Mann–Whitney *U*‐test, or *χ* ^2^ test was used to compare the baseline characteristics of the PS and control patient groups. The Mann–Whitney *U*‐test was also used to compare the RAR parameters (IS, IV, RA, L5, M10) between the PS and control groups of patients. To examine the correlation between the RAR parameters and the scores on the MDS‐UPDRS in the PS group, Spearman's rank correlation coefficients were calculated. To identify factors of the RAR parameters associated with PS, binomial logistic regression analysis was performed with the elderly group (dummy variable, 0 = elderly group, 1 = PS group) as the reference group for the dependent variable, and age, sex and RAR parameters as independent variables. The RAR parameters were converted by scaling (IS × 100, IV × 100, RA × 100, L5/100, M10/100) and then entered as independent variables. Model *χ* ^2^ values were used to determine the adaptation of the regression model, and the Hosmer–Lemeshow test was used to verify the goodness of fit of the regression models. In addition, a discriminant accuracy rate was calculated to determine how accurately the results estimated by the regression model discriminated between the two groups. Finally, activity plots were generated for two cases (Case A and Case B) of the PS group in which the RAR patterns were characterised. The statistical analyses were performed using SPSS version 29.0 (IBM SPSS statistics), and the statistical significance level was set at 5%.

## 3 Results

Table 1 lists the demographic data. The mean age ± standard deviation (SD) was 74.8 ± 6.2 years in the PS group and 75.5 ± 7.7 years in the control group. The PS group comprised 16 patients with PD, 1 patient with MSA, 2 patients with PSP and 1 patient with CBD. The H&Y severity classification was II in 4 patients, III in 12 patients and IV in 4 patients, with III being the most common class. The median (interquartile range) MDS‐UPDRS score was 9.5 (7) for Part I, 13.5 (14) for Part II, 17.5 (23) for Part III and 0 (4) for Part IV in the PS group. Significantly lower values of IS, RA and M10 or higher values of IV were observed in the PS group (*p* < 0.05) as compared with the control group. However, there was no significant difference in the L5 between the two groups (Table 1).

**TABLE 1:** Demographic data.

	PS group (*n* = 20)	Control group (*n* = 20)	
	Mean ± SD	Mean ± SD	*p*
Age (years)	74.8 ± 6.2	75.5 ± 7.7	0.74
Gender (male/female, *n*)	10/10	8/12	0.53
Diagnostics (PD/MSA/PSP/CBD, *n*)	16/1/2/1	—	N/A
H&Y (II/III/IV, *n*)	4/12/4	—	N/A
History of present illness (years)	8.1 ± 6.0	—	N/A
CDR (scores)	0.8 ± 0.7	0.1 ± 0.2	< 0.001**
FIM (scores)	108.9 ± 9.7	126 ± 0	NaN

	Median (IQR)	Median (IQR)	*p*
MDS‐UPDRS‐Part I (scores)	9.5 (7)	—	N/A
MDS‐UPDRS‐Part II (scores)	13.5 (14)	—	N/A
MDS‐UPDRS‐Part III (scores)	17.5 (23)	—	N/A
MDS‐UPDRS‐Part IV (scores)	0 (4)	—	N/A
IS	0.60 (0.28)	0.68 (0.10)	0.04*
IV	1.24 (0.70)	0.89 (0.36)	< 0.001**
RA	0.76 (0.29)	0.89 (0.08)	< 0.001**
L5 (count)	1270 (1130)	1081 (654)	0.37
M10 (count)	8064 (6590)	14826 (4297)	< 0.001**

*Note:* *p < 0.05, **p < 0.01. The *p* value indicates the result of the independent two‐group *t*‐test, Mann–Whitney *U* test, or *χ*2 test.

Abbreviations: CBD, corticobasal degeneration; CDR, clinical dementia rating; FIM, the Japanese version of the Functional Independence Measure; H&Y, Hoehn and Yahr; IQR, interquartile range; IS, inter‐daily stability; IV, intradaily variability; L5, average activity count for the least active 5 consecutive hours in a 24‐h day; M10, average activity count for the most active 10 consecutive hours in a 24‐h day; MDS‐UPDRS, Movement Disorder Society‐Unified Parkinson's Disease Rating Scale; MSA, multiple system atrophy; N/A, not applicable; NaN, not a number; PD, Parkinson's disease; PS, Parkinson's syndrome; PSP, progressive supranuclear palsy; RA, relative amplitude; SD, standard deviation.

Table 2 shows the results according to the Spearman's rank correlation coefficients, which showed a negative correlation between the RA and score on part I of the MDS‐UPDRS (*r* = −0.51, *p* < 0.05). Additionally, the results of the binomial logistic regression analysis with the likelihood ratio (forward selection) are shown in Table 3. Model I showed a significant association with the M10 value (odds ratio, 0.96; 95% confidence interval [95% CI], 0.94–0.99; *p* = 0.002). In addition, Model II showed a significant association with IV (odds ratio, 1.04; 95% CI, 1.01–1.07; *p* = 0.004). The regression equation for each model was significant (*p* < 0.01) and the results of the Hosmer–Lemeshow test indicated a good fit of the models (*p* > 0.05). The discriminant accuracy of the regression model was 87.5% for Model I and 67.5% for Model II.

**TABLE 2:** Result of Spearman's rank correlation coefficient in the PS group.

Variable	1	2	3	4	5	6	7	8	9
1. Age									
2. UPDRS‐I	−0.03								
3. UPDRS –II	0.06	−0.77**							
4. UPDRS‐III	0.30	0.50*	−0.67**						
5. UPDRS‐IV	−0.07	0.19	0.34	0.29					
6. IS	0.28	−0.43	−0.13	−0.11	0.10				
7. IV	−0.16	0.33	0.06	−0.10	−0.29	−0.62**			
8. RA	0.19	−0.51*	−0.33	−0.05	0.06	0.81**	−0.71**		
9. L5	0.02	0.21	0.28	0.16	0.13	−0.42	0.20	−0.68**	
10. M10	0.16	−0.31	0.05	0.10	0.39	0.47**	−0.72**	0.41	0.31

*Note:* **p < 0.01, *p < 0.05, Spearman's rank correlation coefficient. Values in table indicate correlation coefficients.

Abbreviations: IS, inter‐daily stability; IV, intradaily variability; L5, average activity count for the least active 5 consecutive hours in a 24‐h day; M10, average activity count for the most active 10 consecutive hours in a 24‐h day; MDS‐UPDRS, Movement Disorder Society‐Unified Parkinson's Disease Rating Scale; RA, relative amplitude.

**TABLE 3:** Result of binomial logistic regression analysis.

		*β*	SE	Odds ratio	95% CI		*p*
Model I	M10/100	−0.04	0.01	0.96	0.94	0.99	0.002**
	Constant	4.86	1.63	128.35			0.003**
Model II	IV × 100	0.04	0.02	1.04	1.01	1.07	0.004**
	Constant	−4.66	1.58	0.01			0.003**

*Note:* Reference group: the control group. Independent variables included in Model I: age, sex, IS × 100, IV × 100, RA × 100, L5/100, M10/100. Model I: Model *χ* ^2^ value *p* < 0.01, Hosmer–Lemeshow test *p* = 0.26, discriminating accuracy = 87.5%. Independent variables included in Model II: Age, Gender, IS × 100, IV × 100, RA × 100. Model II: Model *χ* ^2^ value *p* < 0.01, Hosmer–Lemeshow test *p* = 0.10, discriminant power = 67.5%.

Abbreviations: 95% CI, confidence interval; IV, intra‐daily variability; M10, Average activity count for the most active 10 consecutive hours in a 24‐h day; SE, standard error; *β*, coefficient.

**FIGURE 1:**
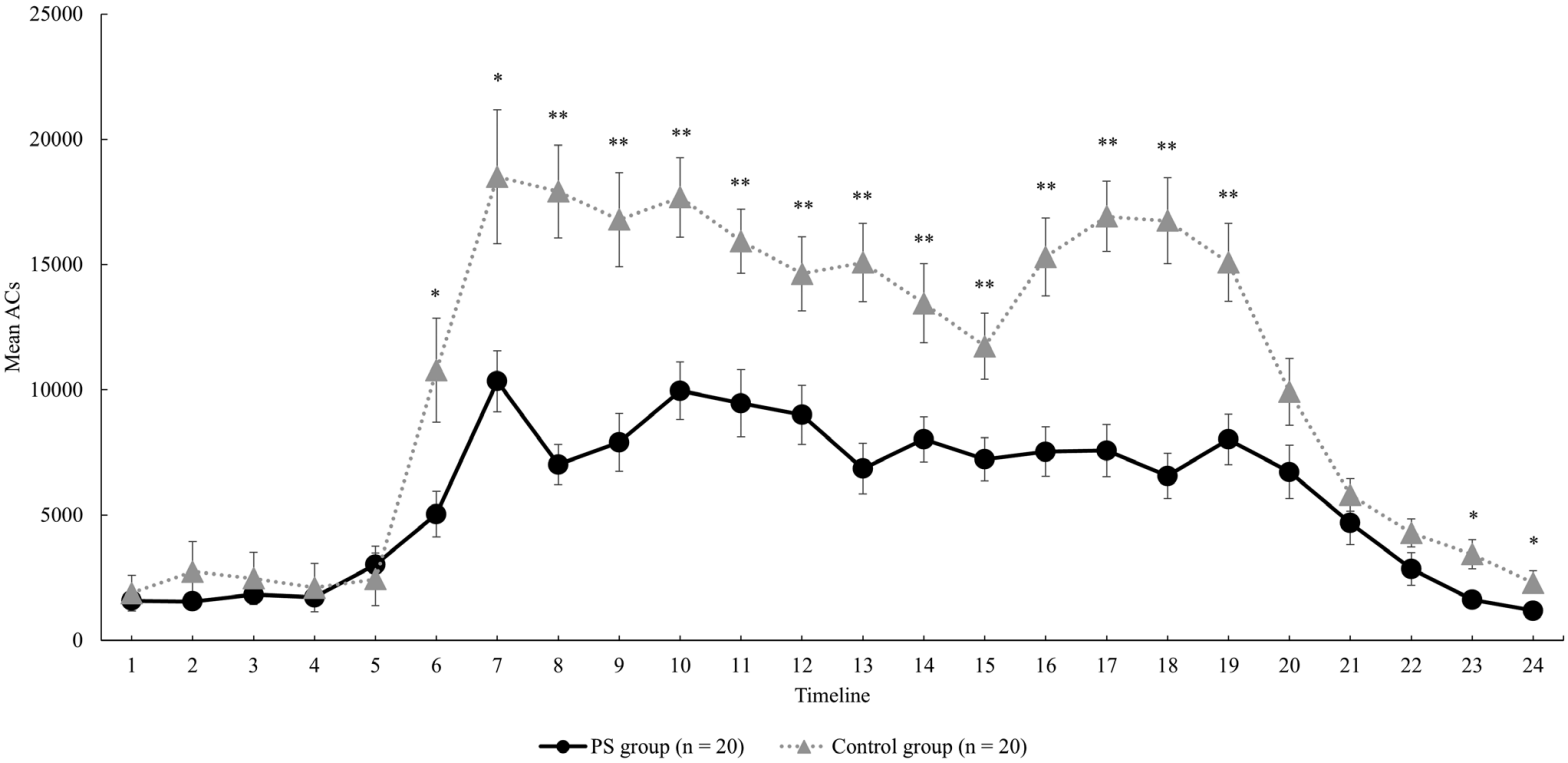
Comparison of Activity Plots between the Groups. *p < 0.05, **p < 0.01, unpaired *t* test. Each error bar indicates the Standard Error of the Mean (SEM). The horizontal axis indicates the timeline from 0:00 AM to 23:59 PM (e.g., timeline 1 is 0:00–0:59 AM, timeline 24 corresponds to 23:00–23:59 PM). AC, activity counts.

**FIGURE 2:**
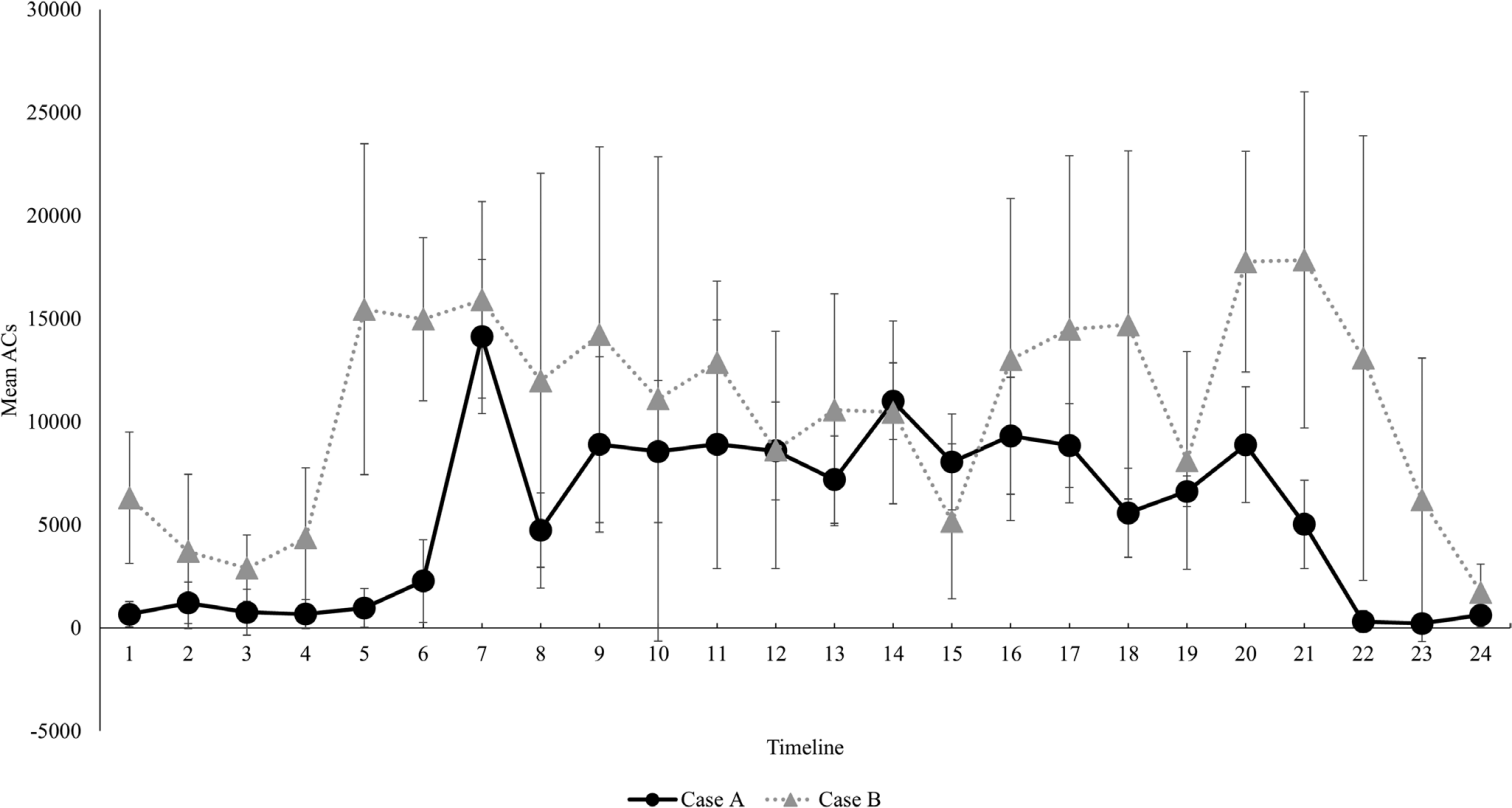
Good (Case A) and Poor (Case B) RAR parameters in the PS Group. The basic information for each case is shown below in the following order: Diagnostics, age (years), gender, H&Y severity classification, History of present illness (years), CDR (scores), FIM (scores), MDS‐UPDRS (scores), IS, IV, RA, L5 (count) and M10 (count). Case A: PD, 74 years old, female, H&Y severity classification II, 1.8 years, CDR = 1.0, MDS‐UPDRS Part I = 10, Part II = 7, Part III = 11, Part IV = 0, FIM = 100, IS = 0.76, IV = 1.03, RA = 0.87, L5 = 610, M10 = 8945. Case B: PD, 74 years old, female, H&Y severity classification III, 17.2 years, CDR = 2.0, MDS‐UPDRS Part I = 22, Part II = 27, Part III = 48, Part IV = 7, FIM = 101, IS = 0.39, IV = 1.26, RA = 0.47, L5 = 4178, M 10 = 11584.

Finally, a comparison of the activity plots in the groups is also shown in Figure 1. In regard to the averages by the time period, the PS group showed significantly lower physical activity levels except in the timelines 1 to 6 (00:00–05:59 h), timeline 21 (20:00–20:59 h) and timeline 24 (23:00–23:59 h) as compared with the control group (Figure 1). Figure 2 shows cases of the PS group with a good RAR pattern (Case A) and with a poor RAR pattern (Case B). The RAR parameters for Case A and Case B were as follows: IS, 0.76 and 0.39, respectively; IV, 1.03 and 1.26, respectively; RA, 0.87 and 0.47, respectively; L5, 610 and 4178, respectively; and M10, 8945 and 11 584, respectively. The IS and RA were lower and IV was higher in case B. In addition, the physical activity level during the nighttime sleep period, L5, was higher in Case B than in Case A, indicating a difference even in the resting state at night.

## 4 Discussion

From the results of the binomial logistic regression analysis in this study, the M10 value was extracted as a relevant RAR indicator for Model I and the IV value for Model II, revealing the characteristics of the rest and activity patterns of the PS group. The M10 value extracted as a relevant factor in model I reflects the level of physical activity during the day. In a previous study that partially supports the results of the present study, Whitehead et al. compared the circadian rhythm parameters of 50 PD patients (mean age ± SD, 73.4 ± 7.5 years; male, 76%) and 29 healthy elderly subjects (mean age ± SD, 70.9 ± 5.6 years; male, 69%) and reported that the circadian rhythm parameters of the 50 PD patients were similar to those of the 29 normal elderly subjects. The results showed a decrease in the RA between rest and activity states (low RA value) and an increase in diurnal variability (high IV value), independent of age or cognitive ability [18]. In particular, they indicated that the decrease in RA reflected a decrease in average daytime physical activity level (low M10 score) and a nighttime arousal state (high L5 score), which may partially support the results extracted by the regression model in this study. In addition, Hilten et al. investigated the effects of physical activity and daytime fatigue in 65 patients (mean age 66.6 years) with mild to moderate PD, corresponding to stage 2.9 ± 0.8 on the H&Y classification (mean ± SD) as compared with those in 68 normal subjects (mean age 66.9 years) [19]. The results showed that the physical activity levels in cases with H&Y classification stage III and IV/V groups were significantly lower than those in healthy subjects (*p* < 0.0001), while those in stage I/II patients did not differ significantly from the levels in healthy subjects [19]. The frequency distribution of the H&Y classification in the PS group in this study showed that stage III was the most common, similar to the subjects in the aforementioned study, which we believe supports the results of this study. In addition, a comparative study by the total UPDRS score has also been performed, which revealed a significant decrease in the physical activity levels during the day, especially in the group with total UPDRS scores of 51–87 (*n* = 15) [19]. In this study, the MDS‐UPDRS scores were calculated for each part and the mean total score was 40.5. Although the PS group had milder disease, on average, than in the aforementioned study, we found a similar decrease in the daytime physical activity levels (lower M10 score) in a two‐group comparison (Table 1) with the control group in the present study. Furthermore, a comparison of the activity plots by time of day showed that the PS group had significantly lower physical activity levels, except in timelines 1–6 (00:00–5:59 h), 21 (20:00–20:59 h) and 24 (23:00–23:59 h).

Next, the IV values extracted as relevant factors in model II indicate the intermittency of rest and activity patterns. Prusynski et al. examined the relationship between daytime napping, nighttime sleep and physical activity in 25 PD patients and 27 healthy elderly subjects, and reported that the relationship between daytime napping and physical activity levels was significantly higher in PD patients than in the control subjects [20]. The results showed that the relationships among daytime napping, nighttime sleep and physical activity were significantly higher in PD patients than in the control subjects. In one study, daily physical activity was converted into metabolic equivalents (METs), as follows: (1) vigorous activity (≥ 6 METs), (2) moderate activity (3–6 METs), (3) light activity (1–3 METs) and (4) sedentary (< 1 MET). This study reported that PD patients slept 75 min less per day (*p* < 0.01) and were less physically active in all the MET categories and spent 32% more time being sedentary (*p* < 0.001) as compared with healthy elderly subjects, suggesting intermittent rest and activity patterns [20]. Niwa et al. also monitored the resting activity using an actigraph in 27 idiopathic PD patients and 30 age‐matched controls and found that PD patients were less physically active when getting out of bed, but more physically active when lying in bed [21]. Although there is little evidence to directly support the fragmented patterns of RAR in PD patients, factors that may influence the high fragmentation of RAR in the PS group in this study have been suggested to include a supine or sedentary lifestyle during the day and a consequent shortened sleep duration at night.

Part I of the MDS‐UPDRS assesses non‐motor symptoms of daily living in PD patients, including cognitive impairment, hallucinations and psychiatric symptoms, depressed mood, anxiety, apathy, symptoms of dopamine dysregulation syndrome, sleep problems, daytime sleepiness, pain and other sensory abnormalities, urinary problems, constipation, dizziness and fatigue. A systematic review by Khan et al. (7 articles, 980 PD patients analysed) reported that the low activity state and sedentary time (1.5 h) in PD patients were significantly correlated with the low activity state and sedentary time (1.5 h). A recent 5‐year follow‐up study of Timblin et al. reported the existence of associations also among hypoactivity, depression and cognitive impairment [22]. Our findings of a low RA score in the PS group in the present study reflected a state of low activity during the day or mid‐afternoon and awakening at night, the negative correlation between the RA and scores for MDS‐UPDRS Part I (non‐motor symptoms), and this finding was supported by the aforementioned reports of Khan et al., and Timblin et al. used the Geriatric Depression Scale (GDS) and Parkinson's Disease Questionnaire (PDQ)‐39 to assess the non‐motor symptoms in PD patients, but not the more comprehensive assessment by Part I of the MDS‐UPDRS [23]. Although this represents a limitation in terms of the structural conceptual validity of the MDS‐UPDRS utilised in this study, MDS‐UPDRS Part I is specifically designed to provide a comprehensive assessment of non‐motor PD symptoms, rather than the standard practice of scoring subscores for individual symptoms separately [11]. Additionally, MDS‐UPDRS Part I includes questions about daily living, focusing on non‐motor aspects and captures the daily experiences of individuals with non‐motor symptoms [24]. Therefore, while the reliability and validity of the MDS‐UPDRS are well‐established in its four‐part multidimensional assessment, it remains challenging to determine in this study which domains of MDS‐UPDRS Part I primarily contribute to the association with RA scores. It can thus be concluded that the findings of this study offer a broader perspective on non‐motor symptoms in the PS group and highlight areas for further research. On the other hand, Brooks et al. found a significant association of the scores for Part II (*p* = 0.01), Part III (*p* = 0.02) and total score (*p* = 0.01) of the MDS‐UPDRS with the physical activity levels in 13 PD patients categorised into stages I–III of the H&Y classification [25]. In addition, a large cohort study of PD patients (*n* = 699) and controls (*n* = 1959) by Nimwegen et al. reported an association between decreased daily physical activity levels and decreased walking ability, increased disability in ADL and increased disease severity in PD patients [1]. These findings of the rest and activity patterns and physical activity levels being associated with the severity of motor symptoms in PD differ from those of the present study. The present study showed that the degree of balance between rest and activity, as indicated by the RA levels, was associated with the severity of the non‐motor symptoms. However, the number of cases was small, and we did not examine which specific non‐motor symptoms the RA levels were associated with.

Case A showed peak activity in the morning, followed by a constant amount of activity and a relatively good night's sleep, while Case B showed a ‘mountain’ waveform of increased activity and a ‘valley’ waveform of rest. These waveforms, with repeated peaks and valleys, indicate intermittency of physical activity. The IS value was low, indicating a large diurnal variation, and an irregularity of the life cycle was evident. In addition, as seen from the RA scores, the patients were generally hyperactive and did not get enough sleep at night, indicating a poor balance between rest and activity. In the MDS‐UPDRS assessment, Case B scored high for all parts, indicating a high severity of disease. Whitehead et al. found that those with hallucinations had lower diurnal stability (*p* < 0.01) and significantly greater nocturnal activity (*p* < 0.05) than those without hallucinations [18], and also significantly lower RA of activity (*p* < 0.05) than those without hallucinations [19], findings that support the characteristics of Case B. Thus, the RAR patterns differ from case to case, and lifestyle advice and intervention should be tailored to each individual based on appropriate assessment.

Several limitations of this study should be mentioned. Firstly, Perez‐Lloret et al. studied the RARs in PD patients (*n* = 60) receiving pharmacotherapy, including levodopa and dopamine agonists (dopamine agonists) and found that the same pharmacotherapy was significantly associated with early morning awakening time (odds ratio 4.0, 95% CI; 1.15–14.13) [26]. The present study included patients who were relatively stable on pharmacotherapy, but the effect of pharmacotherapy was not examined. In addition, Bryant et al. reported that a history of falls and fear of falling may influence a low activity status and limitations in ADL in the PS group [27] suggesting the influence of falls and the associated psychological aspects on the physical activity levels during the day. The heterogeneity of diseases in the PS group is the second issue. A recent study and meta‐analysis reported the following heterogeneity in symptoms among PD, MSA, PSP and CBD; PD: Motor symptoms include tremor, muscle rigidity, motor lability and difficulty maintaining posture. Non‐motor symptoms include a decreased sense of smell, constipation, depression and cognitive dysfunction. MSA: Motor symptoms include cerebellar ataxia (MSA‐C) and parkinsonism (MSA‐P). Autonomic symptoms such as dysuria and orthostatic hypotension are also observed. PSP: Motor symptoms include vertical nystagmus, early falls and postural instability. Non‐motor symptoms consist of cognitive dysfunction and language impairment. CBD: Motor symptoms include asymmetric muscle rigidity, ataxia and tremor. Non‐motor symptoms include cognitive dysfunction and speech impairment [28, 29]. Due to the small number of subjects and the bias toward subjects with PD in the present study (PD/MSA/PSP/CBD, *n*; 16/1/2/1, Table 1), the heterogeneity in cognitive and neuropsychiatric profiles within the PS group warrants further investigation. On the other hand, there is limited information available regarding RAR characteristics in PS, and our preliminary results suggest potential features of RAR parameters in diseases presenting with Parkinson's syndrome. Third, a significant research limitation is the difficulty in generalising the regression models estimated in this study. The sample size was determined based on Peduzzi et al.'s criterion of ‘10 or more events per explanatory variable’ for logistic regression analysis [30]. However, the sample size estimate for the logistic regression analysis, calculated using G*Power version 3.1.9.7, indicates that 4846 subjects would be required to detect a clinically significant effect with the following parameters: number of groups = 2, α = 0.05, power = 80% and odds ratio = 2.0 for binomial logistic regression [31]. Considering the previously mentioned criteria for determining sample size in logistic regression analysis, it is important to note that the regression model applied in this study can be considered generalizable. Furthermore, since PD cases make up the majority of the participants, the findings are more likely to predominantly reflect the characteristics of PD rather than those of PS as a whole. This should be carefully considered when interpreting the results of the study.

## 5 Conclusion

Our results showed that the IS, RA and M10 values were significantly lower, while the IV values were significantly higher in the PS group than in the control group of community‐dwelling elderly persons. Among these RAR parameters, the M10 and IV were the factors that most characterised the rest and activity patterns of the PS group. Furthermore, the RA levels were found to be associated with the non‐motor symptoms in disease‐specific assessments. These results suggest the need to increase the physical activity levels while minimising intermittency of the rest and activity patterns, and to focus on non‐motor as well as motor symptoms in the rehabilitation of patients with PS.

## Conflicts of Interest

The authors declare no conflicts of interest.

## Data Availability

The data that support the findings of this study are available on request from the corresponding author. The data are not publicly available due to privacy or ethical restrictions.
